# Retrospective Cohort Study of 221 Cases of Epidural Per-Cutaneous Peri-Neural Release (Adhesiolysis)

**DOI:** 10.3390/neurosci7020033

**Published:** 2026-03-06

**Authors:** Yasin Said Almakadma, Jouhara Jouhar, Abdulaziz Farooq, Tahani Albatarni

**Affiliations:** 1 Aspetar Orthopedic and Sports Medicine Hospital, Doha 29222, Qatar; yasin.almakadma@aspetar.com (Y.S.A.); jouhara.jouhar@aspetar.com (J.J.); mohammed.farooq@aspetar.com (A.F.); 2 Weil-Cornell Medical College, Doha 24144, Qatar; 3 College of Medicine, Qatar University, Doha P.O. Box 2713, Qatar

**Keywords:** radiculopathy, epidural adhesiolysis, patient reported outcome measures, low back pain, peri-neural release

## Abstract

Background: Lower limb (LL) and low back Radicular Syndromes (RSs) may result from discopathy of the lumbo-sacral spine. Consistent benefits are reported from Epidural Adhesiolysis (EA). Aim: To evaluate clinical and European Quality of Life items (EQ-5D) of Peri-Neural Release interventions (PNR, a modified approach and terminology for EA) among patients diagnosed with lumbo-sacral discopathy associated radicular syndromes. Methods: A retrospective study was conducted by retrieving records of patients e treated by PNR for low back and lower limbs pain between January 2018 and December 2024. Eligible patients were adults who were diagnosed with lumbo-sacral discopathy, stenosis, or Post Lumbar Surgery Syndrome (PLSS). Data on Patient-Reported Outcome Measures (PROMs) adopting the European Quality of Life five items (Euro-QoL 5D) that includes self-ratings of mobility, active daily living, self-care, pain and discomfort, anxiety and depression) was collected before the procedure and on subsequent follow-up visits. Other clinical outcomes included numerical pain rating scales (NRSs), sleep quality, time to pain during activity, and self-reported health scores. Results: A total of 221 patients were included in this analysis. Of these, 56.6% were female, with a mean age of 45.1 ± 14.7 years. In total, 50.2% of patients underwent PNR alone, followed by 28.1% who underwent PNR balloon decompression neuroplasty. Of the remaining patients, 7.2% underwent epiduroscopic PNR, 6.3% PNR combined with annuloplasty (biacuplasty) and 8.1% underwent PNR combined with nucleoplasty. Significant improvements were observed across all EQ-5D and NRS (*p* < 0.001) at follow-up assessments without major complications. The interventions were associated with a decrease in NRS from 7.9 to 3.1, and an increase in the duration of pain-free activity (walking, standing, sitting) (*p* < 0.001). Self-reported overall health scores improved from 53.9 ± 18.4 to 81.1 ± 15.1. In terms of complications, two patients reported post-operative headache. The remaining side effects included coccydynia at the site of intervention, resolving with application of non-steroid anti-inflammatory topicals and self-resolving lower limb numbness in five cases. Conclusions: The presented data suggest that PNR—whether performed alone or in combination with adjunctive intradiscal procedures—is a safe intervention, and is associated, in the majority of patients, with substantial pain relief and improvement in EQ-5D both in the short- and long-term follow-up.

## 1. Introduction

Lower limb (LL) and low back radicular syndromes (RSs) refer to a group of conditions where nerve roots are compressed or irritated, leading to radiating pain, sensory disturbances, and functional limitations in the lower back and legs. Thus, the resulting lumbar radiculopathy is a common manifestation caused by disc herniation, spinal stenosis, or postoperative complications such as epidural fibrosis following lumbar spine surgery [1,2,3]. These conditions are highly prevalent and represent a leading cause of chronic pain and disability globally as reported from different countries, populations and age groups [4,5,6,7].

Chronic inflammatory processes, microvascular impairment, adhesions, and fibrosis in the epidural peri-neural medium are potentially responsible for the refractory nature of symptoms reported by patients suffering from chronic LBP and radiculopathy of LL [8]. These changes restrict nerve root mobility, impair perineural circulation, and perpetuate pain pathways despite conservative therapy [9]. Interventions such as Epidural Adhesiolysis (EA) aim to reverse these mechanical and biochemical contributors, thereby restoring function and improving quality of life [10,11].

Physiotherapy remains a cornerstone of conservative management, particularly when considering non-specific LBP without radiculopathy. Modern approaches favour active versus passive modalities in the case of non-specific LBP [12]. Its benefit seems more questionable in patients with radiculopathy even when combined with simple interventions such as Transforaminal Nerve Root Injections (TFNRIs). In surgical patients, in the short term, pre-op education helped to reduce kinesiophobia post-operatively although it did not affect post-operative pain levels or reported disability [13]. Conversely, TFNRI alone was not more cost-effective than physiotherapy as reported by ter Meulen et al., although their study was related to acute sciatica [14]. A six-month financial value evaluation was in favour of microdiscectomy when compared to conservative approaches including 17 “successful” injections out of 46 patients [15]. In their extensive review, “Epidural Interventions in the Management of Chronic Spinal Pain: American Society of Interventional Pain Physicians (ASIPP) Comprehensive Evidence-Based Guidelines”, Manchikanti et al. concluded to level II with a moderate to strong recommendation for per-cutaneous EA in patients who failed to respond to conservative therapy (including physiotherapy) and to fluoroscopically guided epidural steroid injections [16].

When conservative management fails, treatment options diverge into surgical and interventional approaches. Surgical decompression and discectomy are often proposed in cases with progressive neurological deficits or severe pain. However, studies show that even after spinal surgery, 10–40% of patients may continue to suffer from neuropathic pain resulting in PLSS (Post Lumbar Surgery Syndrome), otherwise described as “persistent spinal pain syndrome” (PSPS) Type II [2].

Non-surgical, interventional techniques, such as EA, have emerged as effective alternatives, particularly for patients with chronic lumbar radiculopathy who have no motor deficit, are poor surgical candidates or those wishing to avoid surgery. Techniques like balloon decompression, contralateral retrodiscal transforaminal approaches, and targeted neuroplasty have shown promising results in reducing pain and improving function [17,18,19]. While surgical decompression may offer faster short-term relief, with success rates reported between 70 and 90%, the risks of recurrence, scar tissue formation, and long-term neuropathic pain remain high, requiring long-term management. On the other hand, as an intermediate approach to RS, interventional techniques are effective in relieving symptoms in 50–80% of patients, less likely to result in serious complications and offer the potential for repeatability if needed [3,19,20,21,22,23]. Among these, benefits from EA exceed those reported from steroid TFNRIs [24].

In addition to EA, discopathy-related symptoms may benefit from other minimally invasive interventional techniques, such as nucleoplasty (aiming to reduce intradiscal pressure), and annuloplasty (biacuplasty), aiming to improve vertebrogenic and discogenic pain. Basivertebral nerve ablation has the potential to reduce vertebrogenic pain [25]. Compared to open surgery, these techniques offer a less invasive approach with reduced recovery time and potentially fewer complications [26,27].

The objective of this study is to evaluate the clinical outcomes, pain levels and EQ-5D scores of percutaneous Peri-Neural Release (PNR), of which the approach, combination with other techniques and terminology are considered over traditional EA in patients diagnosed with lumbar discopathy associated with lower limb and or lower back radicular syndromes. We will also explore how the time from symptom onset to the procedure and the timing of post-intervention assessment relate to the patient’s response to PNR treatment.

## 2. Materials and Methods

### 2.1. Study Design

This retrospective study analyzed medical records of patients who underwent PNR between January 2018 and December 2024. Data was collected from routinely documented electronic health records and telephone follow-up assessments when applicable.

### 2.2. Setting

The study was conducted at Aspetar Orthopedic and Sports Medicine Hospital. Post-operative assessments occurred at variable time points ranging from 14 to 2314 days (6.3 years).

### 2.3. Participants

Adult patients aged 18 years and above, diagnosed with LBP and/or LL pain related to lumbar discopathy, spinal stenosis, or PLSS with consistent radiological findings at the clinical visit were included if they reported symptoms for over a month and had undergone PNR procedure. Exclusion criteria included active spinal or discal infections, pre-existing psychiatric illness, neurological deficits (such as in multiple sclerosis), lower limb pain primarily from other sources like tendinopathy or severe osteoarthritis. The total number included from operating room records was 230. Nine patients had no follow-up beyond the first week post-operatively and were unreachable afterwards.

### 2.4. The Procedures

#### 2.4.1. Indications

PNR, either alone or in combination with other interventions such as balloon decompression neuroplasty, intra-discal annuloplasty (biacuplasty), nucleoplasty, or epiduroscopy, was offered to patients with low back pain with or without leg pain who were refractory to conservative or previous treatments for at least one month, provided that clinical and radiological findings were consistent. PNR with or without balloon decompression was the preferred option if the patient had multi-level discopathy (>2 levels). If difficulty passing through the epidural space was expected or encountered during the intervention, BdC was performed. Epiduroscopy was performed in patients with previous spine surgery. Biacuplasty was proposed if the MRI images showed annular tears but without definite canal stenosis. Nucleoplasty was chosen if protrusion causing nerve route impingement was confirmed and consistent with patient symptoms.

All patients underwent a detailed assessment of the condition history and clinical examination including basic neurological assessment (motor and sensory topography in particular). Informed surgical consent was obtained as per hospital policy at the time of booking the procedure, in addition to the initial general consent obtained at the time of booking their first consultation, voluntarily allowing the use of anonymous data for scientific purposes. All patients were cleared by the Pre-Assessment clinic (Anesthetists and Physicians). The procedures were carried out under sedation-analgesia and local anesthetics in the prone position in fully aseptic conditions and after preoperative antibiotic prophylaxis (Cefazolin 2 g intravascular infusion or Clindamycin 900 mg if allergic).

#### 2.4.2. Steerable Epidural Catheters

The sacral hiatus was accessed using a kit of steerable epidural catheters (SECs), with balloon (Woosung Med-Filtech; Paju-si, Gyeonggi-do, Korea) or without balloon (ST. REED). Radiculography (using Iohexol 300 (5–10 mL)) allowed the identification of ‘filling defects’ which were targeted by means of SEC.

The tip of SEC was directed towards target sites through which Hypertonic saline 3% solution aliquots of 5 mL Hylauronidase (1500 IntUnit in NaCl 0.9% at 300 IntUnit/mL) were infiltrated (Total volume of epidural injectate 30–40 mL). In some cases, where the passage of the device was difficult, very gentle BdC was performed (inflation with 0.3 mL of Iohexol for 5–10 s maximal). Before withdrawing the SEC, MethylPrednisolone at 40 mg or Dexamethasone at 8mg was injected in the epidural space. The SEC (and introducer canula) was withdrawn and examined for any severing or damage. In cases of identified disc bulge with protrusion confirmed in the MRI and correlated with the clinical report, nucleoplasty was performed by Coblation techniques or Quantum Molecular Resonance (QMR). Biacuplasty was performed in the case of back dominant discogenic syndrome, in particular for discopathy with an annular tear.

Patients were then transferred to recovery unit and later to the ward if vitally stable and without motor deficit. Discharge occurred after ensuring stability and ability to empty the bladder. Same day discharge was authorized a few hours later.

All cases were performed by a senior consultant specialized in Interventional Pain and Neuromodulation.

### 2.5. Variables

#### 2.5.1. European Quality of Life Items

Health-related quality of life was self-reported using the EQ-5D-5L questionnaire [28]. This instrument evaluates health status across five dimensions: mobility, self-care, usual activities, pain/discomfort, and anxiety/depression. Participants selected one of five response levels for each dimension, reflecting increasing severity of problems: 1 = no problems, 2 = slight problems, 3 = moderate problems, 4 = severe problems, and 5 = extreme problems or inability to perform the activity. These responses reflected the participant’s health status on the day of assessment [29]. The EQ-5D-5L is a valid and reliable patient-reported outcome measure across various populations [30]. In this study, EQ-5D results were reported at the dimension level only. The EQ-5D visual analogue scale (VAS), which is scored from 0 to 100, was not used because pain intensity was already captured through the EQ-5D pain/discomfort dimension, as well as the Numerical Rating Scale (NRS). VAS administration was not considered optimal given that outcome assessments were conducted by telephone rather than face-to-face.

#### 2.5.2. Quality of Sleep (QoS)

Sleep quality was evaluated based on patient self-reports, aiming to capture sleep disruption associated with symptoms and improvement following treatment. Although multiple sleep evaluation tools exist in the literature, patient self-report is likely the most relevant [31]. Therefore, patients were asked to indicate whether they felt their usual QoS was unaffected (by their symptoms), experienced difficulty falling asleep, frequently woke up or had overall poor QoS (non-repairing sleep).

#### 2.5.3. Activity-Provoked Pain Latency

The time until the onset of pain during standing, sitting, and walking was recorded to assess functional tolerance and activity-related symptom triggers. This delay may reflect the status of daily life as reported in other studies [32]. Activity-provoked pain latency was assessed through patient self-report, referring to a typical day.

#### 2.5.4. Overall Health Score (0–100%)

Patients rated their general health on a percentage scale from 0%, indicating the “worst possible health”, to 100%, indicating the “best possible health”, to provide a subjective, self-perceived evaluation of well-being [33].

### 2.6. Bias

To reduce selection bias, all eligible patients within the specified period were included. Data extraction was standardized, and data extraction was conducted by two independent senior registered nurses. All clinical data were extracted from the electronic records registry.

### 2.7. Follow-Up Assessments

This study analyzed retrospectively collected data. Review of medical records indicated that follow-up assessments were frequently missing, as a substantial proportion of patients did not return for scheduled post-treatment visits. To enhance follow-up data completeness, telephone calls were made to contact patients directly and obtain current self-reported patient-reported outcome measures (PROMs). When multiple follow-up assessments were available for an individual patient, the most recent assessment was selected for analysis to reflect the longest available follow-up and the most sustained post-intervention outcomes.

### 2.8. Confounding

Patient selection was not uniform across intervention subtypes and was driven by routine clinical decision-making. Generally, PNR alone was used for patients with predominantly discogenic pain without significant canal compromise, whereas adjunctive procedures (balloon decompression, nucleoplasty, biacuplasty, or epiduroscopy) were chosen when additional anatomical or clinical features were suspected, such as larger disc protrusions, annular disruption, or epidural adhesions. Consequently, outcomes were reported separately for PNR alone and combination procedures.

### 2.9. Statistical Methods

Data were coded and analyzed using SPSS v21.0. Counts and percentages were used to describe categorical data. The mean and standard deviation were used to describe continuous data. Normality of the data was assessed using the Shapiro–Wilk test. The median and inter quartile range were reported for most parameters in this data that had a Likert scale. For non-normally distributed data, # was used.

For statistical analyses, EQ-5D-5L response levels were treated as numeric ordinal scores and used to quantify changes from pre- to post-intervention. Since outcomes were not normally distributed, EQ-5D-5L scores were analyzed using non-parametric paired tests, primarily the Wilcoxon signed-rank test, to compare pre- and post-intervention values. To account for potential confounding by baseline status, analysis of covariance (ANCOVA) was additionally performed, with post-intervention scores modelled as the dependent variable and corresponding baseline values included as covariates.

The above analysis was repeated for each type of procedural intervention in this study. Patients who reported severe and unable levels in EQ-5D were merged to determine the percentage of patients in those groups. Then we used McNemar’s test to determine the differences in outcomes at post-intervention compared to pre intervention. Cohen’s d was calculated to quantify the magnitude of change between pre- and post-intervention measurements. Effect sizes were interpreted according to conventional thresholds, with values of approximately 0.2 considered small, 0.5 moderate, and 0.8 or greater large. Since, in this study, some follow-up assessments were not collected at standardized time points, the chi-square test for independence was used to study the presence of any trend in outcomes by duration/timing of follow-up assessments (at ≤30 days, 6months and 1 year and above, including those reviewed > 6 years later). A *p*-value < 0.05 was the threshold for statistical significance.

### 2.10. Ethical Considerations

The study was approved by Aspetar’s Institutional Review Board (IRB approval number: E202401069), for both retrospective (the present study) and ongoing prospective cohort study. As a retrospective study, patient signed consent was waived; however, all patients provided written general consent permitting the anonymous use of retrospective clinical data, with the option to opt out, and reaffirmed consent at the time of the specific intervention. All patient data was anonymized and handled in compliance with ethical standards.

## 3. Results

**Figure 1:**
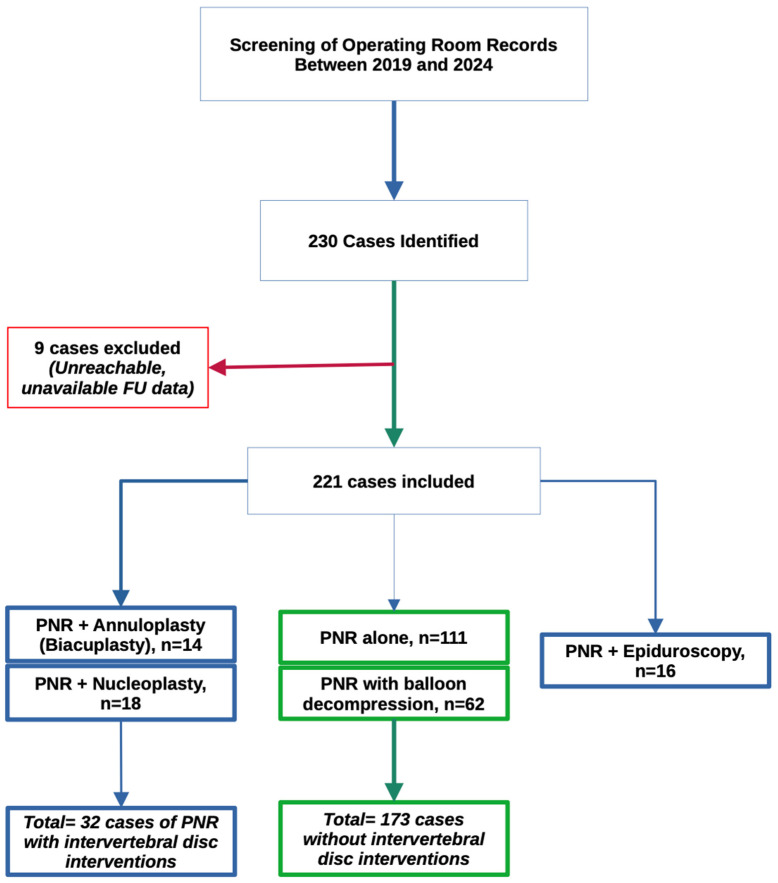
EOne hundred eleven cases of PNR alone and 62 cases of PNR with balloon decompression (BdC), totaling 173 cases. Epiduroscopic PNR, as well as PNR combined with nucleoplasty and annuloplasty (biacuplasty) were performed in 16, 18 and 14 cases respectively.

During the selected study period (2018–2024), data for 230 patients that met the inclusion criteria were available. Nine patients were excluded from the study based on the exclusion criteria. Thus, 221 cases of PNR were retained for the present study as shown in the PRISMA chart (Figure 1).

### 3.1. Demographics

In total, 221 patients were included in this study, of which 56.6% were females. Age ranged from 20 to 79 (mean = 45 ± years). Around 40.7% of patients reported having a physical job, 19.9% were office employees, and 37.1% were without a profession. In total, 65 (29.4%) patients had one or more interventions prior to the PNR, including 51 (23%) who had spinal surgery. All patients had previous or ongoing rehabilitation and pharmacotherapy (NSAIDs, Amitriptyline, Gabapentin or Pregabalin). The average time of reported symptoms before interventions was 2.7 ± 3.0; median: 2 years, ranging from two weeks to 19 years. The duration between intervention and follow-up assessment was 0.95 ± 1.48 years, with a median of 0.19 years (range 0.03–6.34 years). The table shows that patients were distributed across five intervention groups with generally similar baseline characteristics. The epiduroscopic PNR group had a slightly higher mean age (52 ± 10 years), while the other groups had mean ages around the mid-40s. Females represented a larger proportion in most groups. Most patients were either engaged in physical work or had no occupation reported, and the majority had no previous minor interventions before PNR. The duration of symptoms before treatment varied between groups, being longest in the epiduroscopic PNR group and shortest in the nucleoplasty with PNR group. Follow-up assessments were most performed within 30–90 days after the intervention, with some patients followed for longer periods (Table 1).

### 3.2. Pre- vs. Post-Peri-Neural Release (PNR)

This table illustrates the distribution of EQ-5D health status across five domains before and after PNR, categorized as none–mild, moderate–severe, and extreme problems. Prior to PNR, most patients reported moderate to severe impairment across all domains, particularly in pain and discomfort (98.2%), mobility (74.2%), activities of daily living (ADLs) (77.4%), and anxiety/depression (72.4%). All PNR interventions were associated with a marked shift towards none–mild problems categories, observed across all domains. The most notable improvements associated with PNR were seen in mobility, ADLs, and self-care, with over 80% of patients reporting none–mild problems post-PNR. Pain and discomfort also showed substantial improvement, with none–mild problems increasing from 1.8% pre-PNR to 69.7% post-PNR, although a small proportion still reported moderate–severe or extreme pain. Overall, these findings suggest an association with improved health-related quality of life across EQ-5D domains; however, given the retrospective and uncontrolled design, causal inferences regarding the effect of PNR cannot be established (Table 2).

At the end of the follow-up period, all assessed variables including mobility, activities of daily living, self-care, pain, anxiety/depression, quality of sleep, maximum, minimum, and average pain intensity, duration of pain-free activity, maximum pain-free walking, standing, and sitting times, as well as self-reported patient health score showed statistically significant changes from baseline that were observed following PNR across all procedures: Peri-Neural Release (PNR) (Table 3), annuloplasty (biacuplasty) combined with PNR (Table 4), PNR balloon decompression neuroplasty (Table 5), and nucleoplasty combined with PNR (Table 6). At follow-up, none of the patients reported any major complications. Two patients reported post-operative headache. The remaining side effects included coccydynia at the site of intervention, resolving with the application of non-steroidal anti-inflammatory topicals, and self-resolving lower limb numbness in five cases.

**Figure 2:**
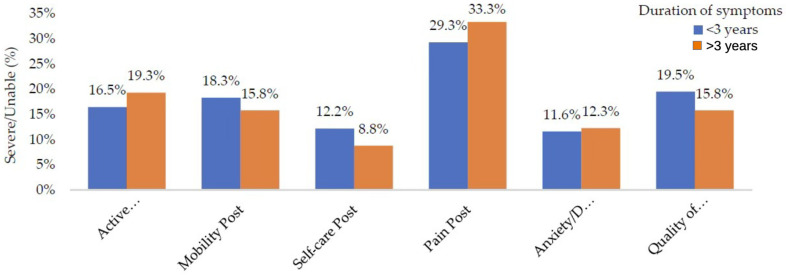
Percentage of severe/unable EQ-5D at follow-up by time intervals between intervention and follow-up assessment among all procedures.

Patients were stratified by symptom duration (<3 years vs. ≥3 years). However, comparative analysis demonstrated that the prevalence of severe/unable EQ-5D domains at follow-up was consistently comparable across both groups, indicating no meaningful differences related to symptom duration (Figure 2).

**Figure 3:**
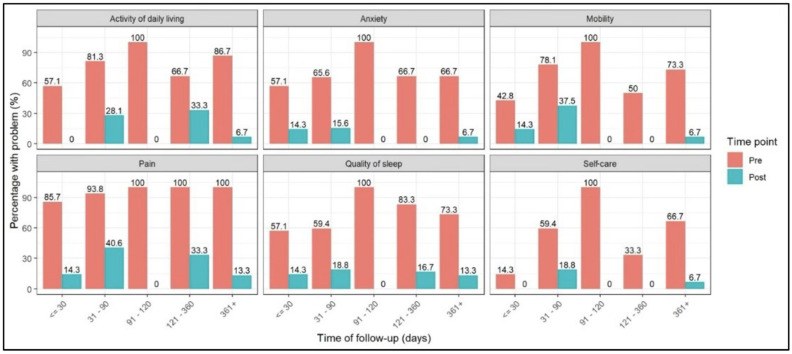
Changes in moderate to severe patient-reported outcomes across variable follow-up intervals before and after PNR balloon decompression neuroplasty (≤30 days, 31–90 days, 91–180 days, 181–360 days, and >360 days).

Moderate to severe mobility impairment ranged from 42.8% to 100% at baseline and from 0 to 37.5% after PNR with BdC. Moderate to severe ADL limitations ranged from 57.1% to 100% before the procedure and from 0% to 33.3% after the intervention, while moderate to severe self-care difficulties ranged from 14.3% to 100% at baseline and from 0% to 6.7% following treatment. Moderate to severe pain/discomfort ranged from 85.7% to 100% before the intervention and declined to 0% to 40.6% after the procedure, representing the most significant improvement that was associated with PNR. Moderate to severe anxiety/depression ranged from 57.1% to 100% pre-procedure and from 0% to 15.6% after the intervention, and moderate to severe sleep disturbance ranged from 57.1% to 100% at baseline and from 0% to 20% following PNR with balloon decompression. Across all follow-up intervals, the prevalence of moderate to severe problems was consistently lower compared with baseline following PNR with balloon decompression (Figure 3).

**Figure 4:**
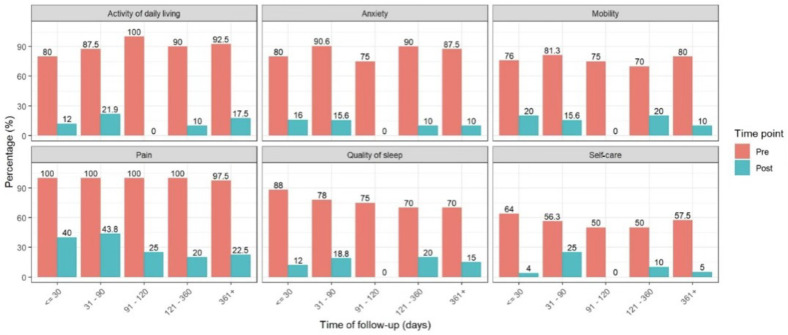
Changes in moderate to severe patient-reported outcomes across variable follow-up intervals before and after Peri-Neural Release (PNR) (≤30 days, 31–90 days, 91–180 days, 181–360 days, and >360 days).

At baseline, prior to PNR alone, moderate to severe impairment was prevalent across all EQ-5D domains. Mobility impairment ranged from 42.8% to 100%, ADL limitations from 57.1% to 100%, and self-care difficulties from 14.3 to 100%. Pain/discomfort was the most affected domain, ranging from 85.7% to 100%, while anxiety/depression and sleep disturbance each ranged from 57.1% to 100%. Following PNR alone, a consistent reduction in the prevalence of moderate to severe problems was observed across all domains and follow-up intervals. Mobility impairment ranged from 0% to 14.3%, ADL limitations from 0% to 33.3%, and self-care difficulties from 0% to 18.8%. Moderate to severe pain declined to 0% to 33.3%, while sleep disturbance and anxiety/depression decreased to 0% to 18.8% and 0% to 15.6%, respectively. This indicates sustained improvement regardless of follow-up timing (Figure 4). Across all available follow-up time points, the prevalence of moderate to severe problems was lower than baseline following the procedure.

## 4. Discussion

PNR, Peri-Neural Release (terminology preferred over EA, Epidural Adhesiolysis), involves advancing a steerable epidural catheter to mechanically disrupt adhesions and deliver targeted pharmacologic agents. Prior work supports its efficacy for refractory radicular pain, with randomized trials showing superior outcomes over conservative therapy [20,34].

In this retrospective cohort of 221 patients, PNR with or without adjunctive minimally invasive disc interventions was associated with significant and clinically meaningful improvements in EQ-5D, duration of pain-free activity, and overall quality of life. The largest relative gains were observed in pain and mobility domains, accompanied by functional increases in walking, standing, and sitting tolerance. The same trend was observed in the reduction in NRS (minimum, maximal, and calculated average). The reports relied on patients’ own report of benefits and their satisfaction about the changes in their functional status rather than on lengthy and complex scoring systems. We acknowledge that these results should be read bearing in mind that the study is retrospective and includes a heterogenous combination of interventions. Nevertheless, in the group of PNR (with and without BdC), the intervention was associated with statistically and clinically meaningful benefits in NRS and PROMs. This remained valid in patients who had their symptoms for less or more than 3 years. In their randomized controlled trial, Manchikanti et al. reported significant clinical improvements from epiduroscopic EA in 80% of patients at 3 months, 56% at 6 months and in 48% at 12 months post-operatively [35]. In patients with or without previous spinal surgery, as reported by Funao et al. and Kim et al., EA was significantly effective in relieving symptoms for up to 6 months post-operatively [36,37].

In our cohort, however, PNR was associated with more sustained benefit than reported with (conventional) EA in the mentioned studies, which could be cautiously explained by the modified approach of PNR and its combined techniques.

EQ-5D dimensions improved significantly across all domains, with the most notable gain in pain/discomfort, followed by mobility. Mean NRS scores decreased from the moderate–severe range to predominantly none or mild post-intervention. These outcomes are comparable to those reported by Manchikanti et al., who observed significant reductions in Oswestry Disability Index (ODI) and pain scores following percutaneous adhesiolysis and NRS improvements at 6 months [38]. Similar benefits were reported after balloon-assisted epidural decompression by Choi et al. [19]. In our present review, PNR was associated with marked improvement across all EQ-5D. Pre-operatively, most patients reported moderate-to-severe impairment in mobility (74.2%), activities of daily living (77.4%), self-care (52.5%), pain and discomfort (98.2%), and anxiety/depression (72.4%). Post-operatively, these rates were significantly reduced, with most patients shifting to none-to-mild impairment: mobility 82.4% vs. 23.1% at baseline, ADLs 82.8% vs. 16.7%, self-care 88.7% vs. 46.6%, and pain 69.7% vs. 1.8%. Mental health status, as reported by patients, evolved very favourably post-intervention: from an initial 77.4% of patients reporting moderate, severe, or extreme anxiety/depression, the great majority (88.2%) reported normal or only mild residual symptoms after treatment.

Our results suggest that PNR is associated with EQ-5D benefits in line with, or exceeding, prior literature reports on similar targeted traditional epidural adhesiolysis techniques. We believe that our modified techniques of targeting sites using steerable devices combined with BdC, annuloplasty or nucleoplasty may have had a role in a better outcome than more conventional catheters.

Physical activities are adversely affected by LBP and LL pain. Walking, standing and sitting time delays without triggering or worsening pain may be useful indicators of progress or improvement of the disease [39,40]. In our cohort, walking tolerance improved from a median of 15 to 45 min, standing from 10 to 30 min, and sitting from 15 to 45 min. These functional gains are consistent with prior studies demonstrating increased activity duration after adhesiolysis-based interventions for FBSS patients, for example. We observed, however, a better and more time-consistent improvement in these indicators within our cohort if compared to other reports [39,40]. This could well be linked to different techniques and combined approaches.

A very high percentage of patients (80%) reported severe to extreme impairment of their QoS. After the PNR treatment, this percentage falls below 20%. Such a good result is likely related to pain reduction, ability to ambulate, and better mental health status.

Patient’s self-reported health scores improved from approximately 54% pre-procedure (mean 53.9 ± 18.4; median 60 (48–70)) to approximately 80% post-procedure (mean 81.1 ± 15.1; median 85 (70–90)), reflecting a substantial and clinically relevant quality of life gain. Similar magnitudes of improvement have been reported after targeted epidural interventions, including adhesiolysis and balloon decompression, where QoL gains were sustained for up to 1 year. The authors believe that this change remains in line with the notable improvement in anxiety and depression status observed in our cohort.

### 4.1. Treatment Techniques and Approaches

#### 4.1.1. Peri-Neural Release (PNR) (Sole Intervention, 11 Cases)

In our cohort, PNR was associated with substantial improvements across all domains: mobility, activities of daily living, and self-care scores approximately halved post-procedure; pain scores decreased markedly, with average intensity dropping from 5.8 ± 1.7 to 1.9 ± 1.7; anxiety/depression scores improved from 3.3 ± 0.8 to 1.5 ± 0.8; and quality of sleep from 2.9 ± 1.1 to 1.4 ± 0.8. Functional gains were notable, with maximum pain-free walking increasing from 23.2 ± 20.4 min to 99.4 ± 397.3 min, standing from 13.7 ± 14.0 to 34.7 ± 21.4 min, and sitting from 20.1 ± 18.4 to 45.5 ± 25.3 min. Self-reported overall health scores improved from 53.9 ± 18.4 to 81.1 ± 15.1, reflecting a meaningful enhancement in quality of life.

#### 4.1.2. PNR Balloon Decompression Neuroplasty (62 Cases)

Balloon-assisted decompression enhances mechanical release and space creation in the epidural canal. Studies have documented significant pain and function gains, especially in adhesive arachnoiditis and spinal stenosis [34]. In our patients, we observed a similar pattern of improvement, with self-reported health scores increasing from 51.9 ± 18.9 (median 50 (40–68)) pre-procedure to 76.5 ± 18.3 (median 80 (70–90)) post-procedure, reflecting an approximate 47% improvement. Associated functional gains were also notable, with maximum pain-free walking time increasing from 22.2 ± 21.6 min to 42.0 ± 29.2 min, standing from 14.0 ± 15.3 to 35.2 ± 25.4 min, and sitting from 16.7 ± 17.2 to 37.6 ± 24.6 min. These benefits continued for up to 12 months in the available follow-up data, indicating both short- and longer-term efficacy of the intervention.

#### 4.1.3. Epiduroscopic PNR (16 Cases)

Although the subgroup of patients who had epiduroscopic PNR is small, our findings confirm previous reports in the literature [41]. Endoscopic visualization allows direct confirmation of adhesion release and targeted medication delivery, associated with reported functional and pain improvements in selected chronic radiculopathy cases.

#### 4.1.4. PNR with Nucleoplasty (18 Cases) or Annuloplasty Biacuplasty (14 Cases)

Nucleoplasty by Coblation or QMR aims to reduce intradiscal pressure while annuloplasty (biacuplasty) is preferred in the presence of annular fissures [26,27,42]. The PNR was combined with nucleoplasty or biacuplasty in patients with documented discal abnormalities consistent with dominant symptoms and suitable for one of the techniques. As PNR targets epidural adhesions, the rationale behind the combination was to address both mechanical and chemical issues believed to produce radicular symptoms. To our knowledge, no previous studies have reported the outcome from the combination of annuloplasty or nucleoplasty with PNR (or adhesiolysis). While surgical techniques have similar discopathy-related indications, nucleoplasty and annuloplasty are less invasive and more cost-effective than surgical discal techniques in addition to yielding comparative benefits, whether by open or percutaneous endoscopy techniques. The same goes for the rate of complications and length of hospital stay [26,43,44]. Patients undergoing PNR combined with nucleoplasty or annuloplasty experienced significant improvements in pain, function, and quality of life. Mobility, activities of daily living, and self-care scores approximately halved post-procedure, while pain scores, including average, maximum, and minimum intensities, decreased substantially. Anxiety and sleep quality also improved. Functional abilities, such as maximum pain-free walking, standing, and sitting time, showed marked gains associated with the procedure. Self-reported overall health scores increased from ~52–58% pre-procedure to ~77–81% post-procedure, reflecting clinically meaningful improvements sustained in available follow-up data.

#### 4.1.5. Effect of Symptom Duration

Kim et al. reported that patients with a shorter symptoms duration prior to intervention (3 years or less) had a better outcome than those who had their symptoms for a longer period of time [45].

In our cohort, we found no meaningful reduction in benefits of PNR in those who had their symptoms for more than 3 years. This could be linked to the type of techniques and combinations giving an additional advantage over that reported in the literature.

### 4.2. Limitations

As a retrospective study, our current cohort review shares limitations related to patient selection, standardization of techniques, absence of comparative sham intervention, and the duration intervals of follow-ups. On the other hand, while there is some missing data, most case notes were available and allowed for a comprehensive analysis of the potential role of PNR in the management of radiculopathic LBP and LL. Several related outcomes were examined across patient-reported quality of life, pain, and functional measures. While no formal adjustment for multiple comparisons was applied, the analyses were conducted to provide a comprehensive descriptive assessment of patient outcomes.

## 5. Conclusions

PNR performed with balloon decompression and PNR without balloon decompression were observed to be associated with significant improvements in pain intensity, functional outcomes, EQ-5D scores, and overall quality of life in patients with chronic radiculopathy. However, given the retrospective design, these findings represent associations rather than causal effects. The effects of other adjunctive procedures could not be adequately explored due to small sample sizes. Further controlled prospective studies are warranted to confirm these associations, compare outcomes between balloon and non-balloon techniques, optimize patient selection, and evaluate the long-term durability and cost effectiveness of these interventions.

## Figures and Tables

**Table 1:** Characteristics of the patients (n = 221).

Performed Intervention	Epiduroscopic PNR	Peri-Neural Release (PNR)	Annuloplasty (Bicuplasty) and PNR	PNR Balloon Decompression Neuroplasty	Nucleoplasty and PNR
Variables					
Age (Mean ± SD)	52 ± 10	44 ± 15	44 ± 12	46 ± 16	44 ± 13
Gender					
Female	13 (81.3%)	57 (51.4%)	10 (71.4%)	35 (56.5%)	10 (55.6%)
Male	3 (18.8%)	54 (48.6%)	4 (28.6%)	27 (43.5%)	8 (44.4%)
Profession					
Missing	1 (6.3%)	0 (0.0%)	0 (0.0%)	0 (0.0%)	0 (0.0%)
Combined (Desk-Office and Physical)	1 (6.3%)	2 (1.8%)	0 (0.0%)	1 (1.6%)	0 (0.0%)
Desk-Office	1 (6.3%)	19 (17.1%)	5 (35.7%)	13 (21.0%)	6 (33.3%)
None	5 (31.3%)	37 (33.3%)	6 (42.9%)	27 (43.5%)	7 (38.9%)
Physical	8 (50.0%)	53 (47.7%)	3 (21.4%)	21 (33.9%)	5 (27.8%)
Previous minor interventions					
No	11(68.8%)	82 (73.9%)	8 (57.1%)	43 (69.4%)	12 (66.7%)
Yes	5 (31.3%)	29 (26.1%)	6 (42.9%)	19 (30.6%)	6 (33.3%)
Duration of symptoms before PNR (Mean ± SD)	45.3 ± 38.5	30.9 ± 35.6	32.1 ± 37.8	33.9 ± 37.5	22.9 ± 22.4
Duration between Intervention and follow-up assessment (days)					
≤ 30	3 (18.8%)	25 (22.5%)	1 (7.1%)	7 (11.3%)	3 (16.7%)
30–90	6 (37.5%)	32 (28.8%)	10 (71.4%)	32 (51.6%)	10 (55.6%)
90–120	0 (0.0%)	4 (3.6%)	1 (7.1%)	2 (3.2%)	1 (5.6%)
120–360	2 (12.5%)	10 (9.0%)	0 (0.0%)	6 (9.7%)	2 (11.1%)
360+	5 (31.3%)	40 (36.0%)	2 (14.3%)	15 (24.2%)	2 (11.1%)

**Table 2:** Distribution of EQ-5D health status domains pre- and post-PNR procedures.

EQ-5D	Status	None–Mild (%)	Moderate–Severe (%)	Extreme (%)
Mobility	Pre	51 (23.1%)	164 (74.2%)	6 (2.7%)
	Post	182 (82.4%)	39 (17.6%)	0 (0.0%)
ADLs	Pre	37 (16.7%)	171 (77.4%)	13 (5.9%)
	Post	183 (82.8%)	37 (16.7%)	1 (0.5%)
Self-Care	Pre	103 (46.6%)	116 (52.5%)	2 (0.9%)
	Post	196 (88.7%)	25 (11.3%)	0 (0.0%)
Pain and Discomfort	Pre	4 (1.8%)	217 (98.2%)	0 (0.0%)
	Post	154 (69.7%)	64 (29.0%)	3 (1.4%)
Anxiety/Depression	Pre	50 (22.6%)	160 (72.4%)	11 (5.0%)
	Post	195 (88.2%)	25 (11.3%)	1 (0.5%)

**Table 3:** EQ-5D, pain and duration of pain-free activity pre- and post-Peri-Neural Release (PNR).

	PRE			POST			
Variable	N	Mean ± SD	Median [IQR]	N	Mean ± SD	Median [IQR]	Cohen’s d
EQ-5D							
Mobility	115	3.3 ± 1.0	4 (3–4)	113	1.7 ± 0.9 *	1 (1–2)	1.36
Active Daily Living	116	3.5 ± 0.9	4 (3–4)	113	1.7 ± 0.9 *	1 (1–2)	1.51
Self-care	115	2.7 ± 1.1	3 (2–4)	113	1.4 ± 0.8 *	1 (1–2)	1.15
Pain	116	4.1 ± 0.4	4 (4–4)	114	2.4 ± 1.2 *	2 (2–4)	1.39
Anxiety/Depression	116	3.3 ± 0.8	3 (3–4)	114	1.5 ± 0.8 *	1 (1–2)	1.74
Quality of Sleep	115	2.9 ± 1.1	3 (3–4)	113	1.4 ± 0.8 *	1 (1–1)	1.25
Pain							
Max. Intensity of Pain	115	7.9 ± 1.3	8 (7–9)	115	3.1 ± 2.4 *	3 (1–5)	1.87
Min. Intensity of Pain	115	3.8 ± 2.5	4 (2–6)	115	0.7 ± 1.3 *	0 (0–1)	1.25
Average Intensity of Pain	116	5.8 ± 1.7	6 (5–7)	115	1.9 ± 1.7 *	2 (1–3)	1.82
Duration of pain-free activity (minutes)							
Maximum time patient is able to walk without pain (minutes)	113	23.2 ± 20.4	15 (5–30)	111	99.4 ± 397.3 **	60 (30–60)	0.19
Maximum standing time (without pain)	112	13.7 ± 14.0	10 (5–15)	111	34.7 ± 21.4 **	30 (15–60)	0.95
Maximum sitting time (without pain)	113	20.1 ± 18.4	15 (5–30)	111	45.5 ± 25.3 **	45 (30–60)	0.97
Self-reported Patient Health Score (0–100)	110	53.9 ± 18.4	60 (48–70)	112	81.1 ± 15.1 **	85 (70–90)	1.27

*: Significantly lower; **: Significantly longer duration/higher score.

**Table 4:** EQ-5D, pain and duration of pain-free activity pre- and post-annuloplasty (biacuplasty) and PNR.

	PRE			POST			
Variable	N	Mean ± SD	Median [IQR]	N	Mean ± SD	Median [IQR]	Cohen’s d
EQ-5D							
Mobility	15	2.9 ± 1.1	3 (2–4)	15	1.5 ± 0.7 *	1 (1–2)	1.20
Active Daily Living	15	3.2 ± 0.9	3 (3–4)	15	1.5 ± 0.9 *	1 (1–2)	1.55
Self-care	15	2.6 ± 1.0	3 (2–3)	15	1.3 ± 0.8 *	1 (1–1)	1.25
Pain	15	4.1 ± 0.3	4 (4–4)	15	2.3 ± 0.9 *	2 (2–3)	1,83
Anxiety/Depression	15	2.7 ± 1.0	3 (2–4)	15	1.5 ± 0.8 *	1 (1–2)	0.85
Quality of Sleep	15	2.8 ± 1.0	3 (3–3)	15	1.6 ± 0.9 *	1 (1–3)	0.98
Pain							
Max. Intensity of Pain	15	7.4 ± 1.4	7 (7–8)	15	3.1 ± 2.0 *	3 (2–4)	1.79
Min. Intensity of Pain	15	4.0 ± 2.6	4 (2–6)	15	0.5 ± 1.4 *	0 (0–0)	1.31
Average Intensity of Pain	15	5.7 ± 1.8	6 (4–7)	15	1.8 ± 1.4 *	2 (1–3)	1.73
Duration of pain-free activity (minutes)							
Maximum time patient is able to walk without pain (minutes)	15	22.4 ± 23.9	10 (5–60)	15	43.3 ± 24.5 **	60 (10–60)	0.77
Maximum standing time (without pain)	15	17.3 ± 19.7	10 (5–15)	15	37.3 ± 20.9 **	40 (15–60)	0.53
Maximum sitting time (without pain)	15	23.7 ± 23.7	10 (5–60)	15	38.0 ± 21.3 **	40 (15–60)	0.54
Self-reported Patient Health Score (0–100)	15	57.7 ± 18.2	60 (50–60)	15	80.7 ± 13.3 **	80 (70–90)	0.85

*: Significantly lower; **: Significantly longer duration/ higher score.

**Table 5:** EQ-5D, pain and duration of pain-free activity pre- and post-PNR balloon decompression neuroplasty.

	PRE			POST			
Variable	N	Mean ± SD	Median [IQR]	N	Mean ± SD	Median [IQR]	Cohens’ d
EQ-5D							
Mobility	65	3.0 ± 1.1	3 (2–4)	64	1.8 ± 0.9 *	2 (1–2)	1.12
Active Daily Living	65	3.2 ± 0.9	3 (3–4)	64	1.8 ± 0.9 *	2 (1–2)	1.30
Self-care	65	2.6 ± 1.2	3 (2–3)	64	1.5 ± 0.8 *	1 (1–2)	0.96
Pain	65	4.0 ± 0.5	4 (4–4)	64	2.5 ± 1.1 *	2 (2–4)	1.33
Anxiety/Depression	65	3.0 ± 1.0	3 (2–4)	64	1.5 ± 0.8 *	1 (1–2)	1.38
Quality of Sleep	65	2.5 ± 1.1	3 (1–3)	64	1.3 ± 0.8 *	1 (1–1)	1.00
Pain							
Max. Intensity of Pain	65	7.3 ± 1.6	8 (6–8)	65	3.5 ± 2.4 *	3 (2–5)	1.37
Min. Intensity of Pain	65	2.5 ± 1.1	3 (1–3)	64	1.3 ± 0.8 *	1 (1–1)	0.84
Average Intensity of Pain	65	3.0 ± 2.4	3 (0–5)	65	1.1 ± 1.7 *	0 (0–2)	1.34
Duration of pain-free activity (minutes)							
Maximum time patient is able to walk without pain (minutes)	65	22.2 ± 21.6	10 (5–30)	64	42.0 ± 29.2 **	40 (16–60)	0.72
Maximum standing time (without pain)	65	14.0 ± 15.3	10 (5–15)	64	35.2 ± 25.4 **	30 (10–60)	0.86
Maximum sitting time (without pain)	65	16.7 ± 17.2	15 (5–25)	64	37.6 ± 24.6 **	30 (15–60)	0.86
Self-reported Patient Health Score (0–100)	65	51.9 ± 18.9	50 (40–68)	64	76.5 ± 18.3 **	80 (70–90)	1.25

*: Significantly lower; **: Significantly longer duration/higher score.

**Table 6:** EQ-5D, pain and duration of pain-free activity pre- and post-nucleoplasty and PNR.

	PRE			POST			
Variable	N	Mean ± SD	Median [IQR]	N	Mean ± SD	Median [IQR]	Cohen’s d
EQ-5D							
Mobility	18	3.1 ± 0.7	3 (3–4)	18	1.8 ± 0.7 *	2 (1–2)	1.74
Active Daily Living	18	2.9 ± 0.9	3 (2–4)	18	1.6 ± 0.7 *	1 (1–2)	0.94
Self-care	18	2.2 ± 0.9	2 (2–2)	18	1.3 ± 0.6 *	1 (1–2)	0.85
Pain	18	4.1 ± 0.2	4 (4–4)	18	2.3 ± 1.0 *	2 (2–3)	1.89
Anxiety/Depression	18	3.2 ± 0.9	3 (2–4)	18	1.3 ± 0.6 *	1 (1–1)	1.67
Quality of Sleep	18	3.1 ± 0.9	3 (3–4)	18	1.4 ± 0.8 *	1 (1–2)	1.48
Pain							
Max. Intensity of Pain	18	7.1 ± 1.7	8 (6–8)	18	2.9 ± 1.7 *	3 (2–4)	1.87
Min. Intensity of Pain	18	3.3 ± 2.6	3 (1–6)	18	0.6 ± 1.1 *	0 (0–1)	1.33
Average Intensity of Pain	18	5.2 ± 1.9	5 (3–7)	18	1.8 ± 1.2 *	2 (1–3)	1.91
Duration of pain-free activity (minutes)							
Maximum time patient is able to walk without pain (minutes)	18	20.7 ± 17.3	15 (9–30)	18	38.4 ± 28.9 **	35 (14–60)	0.55
Maximum standing time (without pain)	18	13.8 ± 13.5	10 (5–16)	18	29.3 ± 22.9 **	25 (9–60)	0.59
Maximum sitting time (without pain)	18	21.7 ± 17.3	15 (9–30)	18	44.2 ± 20.1 **	60 (20–60)	1.20
Self-reported Patient Health Score (0–100)	18	57.5 ± 17.5	60 (50–70)	18	79.8 ± 18.0 **	89 (70–90)	1.12

*: Significantly lower; **: Significantly longer duration/higher score.

## Data Availability

Data related to this study is available from the corresponding author upon request.
